# Effects of FGF2/FGFR1 Pathway on Expression of A1 Astrocytes After Infrasound Exposure

**DOI:** 10.3389/fnins.2019.00429

**Published:** 2019-05-03

**Authors:** Lin-Hui Zou, Ya-Jun Shi, Hua He, Shi-Mei Jiang, Fang-Fang Huo, Xiao-Mu Wang, Fan Wu, Lei Ma

**Affiliations:** ^1^Department of Neurology, Xijing Hospital, Fourth Military Medical University, Xi’an, China; ^2^Frontier Medical Training Brigade, Army Medical University, Changji, China; ^3^Department of Specific Diagnosis, PLA 986 Hospital, Xi’an, China; ^4^Department of Acu-Moxibustion and Tuina, Henan University of Traditional Chinese Medicine, Zhengzhou, China; ^5^Department of Medicine, Yulin Yuyang District Hospital of Traditional Chinese Medicine, Yulin, China

**Keywords:** infrasound, hippocampus, A1 astrocyte, microglial cell, FGF2/FGFR1 pathway

## Abstract

Two types of reactive astrocytes, A1 and A2 astrocytes, are induced following neuroinflammation and ischemia. In this study, we evaluated the effects of the fibroblast growth factor (FGF)2/FGF receptor (FGFR)1 pathway on A1 and A2 astrocytes in the rat hippocampus using double-labeling immunofluorescence following infrasound exposure. A1 astrocytes were induced in the CA1 region of the hippocampus after exposure to infrasound for 3 days. The number of microglial cells was also increased, and we investigated if these might be responsible for the reactivity of A1 astrocytes. Accordingly, expression levels of C3 and Iba-1, as markers of A1 astrocytes and microglial cells, respectively, were both up-regulated in rat hippocampus following infrasound exposure, as demonstrated by western blot. We also explored the effect of the FGF2/FGFR1 pathway on A1 astrocyte reactivity by pretreating rats with FGF2 or the specific FGFR1 antagonist, PD173074. A1 astrocytes were gradually down-regulated by activation of the FGF2/FGFR1 pathway and were up-regulated by inhibition of the FGF2/FGFR1 pathway after infrasound damage. These results further our understanding of the role of reactive astrocytes in infrasound-induced central nervous system injury and will thus facilitate the development of new treatments for these injuries.

## Introduction

Sound waves <20 Hz are referred to as infrasound. Infrasound is generated by numerous environmental factors, including agricultural machinery and industrial processes (Backteman et al., 1983; Bilski, 2017), and has been implicated in various kinds of health damage. For example, infrasound exposure is noted to be harmful in pregnant women (Castelo-Branco and Rodriguez, 1999). Furthermore, human and animal experiments have suggested that prolonged infrasound exposure can damage the central nervous system (CNS), including the hippocampus, cerebellum, limbic-corticular complex, hypothalamus, and cortex (Izmerov et al., 1997; Fei et al., 2000; Yuan et al., 2009; Shi et al., 2013; Cai et al., 2014; Ma et al., 2015). We previously confirmed that infrasound exposure activated astrocytes and induced neuronal apoptosis in the CNS, which subsequently impaired spatial learning and memory abilities (Shi et al., 2013, 2018).

Neuroinflammation and ischemia have been reported to induce two different types of reactive astrocytes, A1 and A2 astrocytes (Zamanian et al., 2012). A1 astrocytes are induced by activated microglia and gain a neurotoxic function, resulting in neuron killing (Liddelow et al., 2017), while A2 astrocytes upregulate many neurotrophic factors and strongly promote neuronal survival and tissue repair (Bush et al., 1999). Double-immunofluorescence labeling with complement component 3 (C3) and glial fibrillary acidic protein (GFAP) had been used to label A1 astrocytes, and double-immunofluorescence labeling with S100a10 and GFAP had been used to label A2 astrocytes (Liddelow et al., 2017).

In this study, we investigated the effects of infrasound on the induction of A1 and A2 astrocytes in the rat hippocampus by double-immunofluorescence labeling with C3 and GFAP, or S100a10 and GFAP, respectively. We also detected microglial cells by immunofluorescence labeling with Iba-1, to investigate their role in the effects of infrasound exposure. We verified the immunofluorescence results by measuring expression levels of C3 and Iba-1 in rat hippocampus.

We previously showed that the fibroblast growth factor (FGF)2/FGF receptor (FGFR)1 pathway inhibited astrocyte-mediated neuroinflammation *in vitro* and *in vivo* after infrasound exposure (Shi et al., 2018), suggesting that the reactivity of A1 astrocytes was related to activation of this pathway. We therefore investigated the role of the FGF2/FGFR1 pathway in infrasound-induced changes of A1 astrocytes in rats pretreated with FGF2 or the selective FGFR1 inhibitor, PD173074 (Mohammadi et al., 1998).

## Materials and Methods

### Infrasound Device

The infrasound radiation laboratory was located at the Center for Radiation, the Fourth Military Medical University, Xi’an, China. The infrasound device included an infrasound chamber and infrasonic signal detection system. Infrasound with a frequency of 16 Hz and a pressure level of 150 dB was used in this study. The frequency and pressure of the infrasound were kept steady during 2 h of animal exposure and monitored using the infrasonic signal detection system.

### Animals

Male Sprague-Dawley rats, weighing 220–250 g, were obtained from the Center of Experimental Animals, Fourth Military Medical University. The rats were maintained in an animal laboratory under controlled conditions at 20–25°C, humidity 50–60%, and a 12-h light/dark cycle, and were provided with free access to rodent chow and water.

The rats were divided randomly into groups as described previously (Shi et al., 2018): control group (no infrasound exposure, *n* = 6), infrasound (IS) exposure groups (exposed to 16 Hz, 150 dB of infrasound for 1, 3, 5, or 7 days, *n* = 6 per group), FGF2 groups (treated with FGF2 for 1, 3, 5, or 7 days, *n* = 6 per group), PD groups (treated with PD173074 for 1, 3, 5, or 7 days, *n* = 6 per group), FGF2+IS groups (infrasound-exposed rats treated with FGF2, *n* = 6 per group), and PD + IS groups (infrasound-exposed rats treated with PD173074, *n* = 6 per group). For FGF2 administration, rats were injected intraperitoneally (i.p.) with 0.1 mg/kg FGF2. For PD173074 administration, rats were injected i.p. with 1.5 mg/kg PD173074. FGF2 was dissolved in saline (Graham and Richardson, 2009) and PD173074 was dissolved in saline containing 12.5% Cremophor EL and 2.5% dimethylsulfoxide (Di Marco et al., 2014). FGF2 and PD173074 were injected everyday. The rats were exposed to infrasound of 16 Hz and 150 dB for 2 h a day.

### Tissue Preparation

Brain slices were obtained as described previously (Melvin and Sutherland, 2010). After infrasound exposure, the rats were anesthetized with 10% chloral hydrate and then perfused sequentially with 200 ml ice-cold saline via a perfusion pump at 30 rpm, 200 ml 4% ice-cold paraformaldehyde (PFA) at 30 rpm, and 200 ml 4% PFA at 4 rpm. The brains were removed, fixed in 4% PFA for 12 h, dehydrated in 30% sucrose solution, embedded in OCT compound, and sectioned transversely into 35 μm-thick slices using a Leica CM 1900 cryostat. These sections were stored in 60% glycerine at −20°C and used for immunofluorescence.

### Primary Astrocyte Culture

Primary cultures of rat hippocampus astrocytes were prepared from neonatal rats. Briefly, hippocampus tissue was minced with forceps and digested with 0.05% trypsin for 5 min. DMEM (Corning, New York, NY, United States) containing 10% fetal bovine serum, 1% glutamine, and 1% penicillin was added to stop the digestion and the supernatant was filtered. After centrifugation at 1000 rpm for 5 min, the cell mass was resuspended in DMEM and streptomycin at 37°C. The culture medium was replaced every 3 days. The purity of the astrocytes was determined by immunostaining with GFAP antibody, as described below. For the experiments, the astrocytes were exposed to infrasound of 16 Hz and 150 dB for 2 h, fixed with 4% PFA for 20 min, and stored for immunofluorescence staining.

### Immunofluorescence

Brain sections or cultured astrocytes on coverslips were blocked with 3% bovine serum albumin (BSA) in PBS containing 0.3% Triton X-100 for 1 h at room temperature (RT). The sections or cells were then incubated with primary antibodies overnight at 4°C. Two antibodies were added simultaneously for double-immunofluorescence staining. The following antibodies were used: rabbit anti-S100a10 (1:100; Novus, Colorado Springs, CO, United States), mouse anti-C3 (1:100; Santa Cruz Biotechnology, Santa Cruz, CA, United States), rabbit anti-Iba-1 (1:800; Sigma, United States), mouse anti-GFAP (1:500; Abcam, Cambridge, United Kingdom), and rabbit anti-GFAP (1:500; Abcam). The samples were then incubated with species-specific secondary antibodies conjugated with Alexa Fluor (1:200; Zhuangzhi, Beijing, China) for 2 h at RT, and nuclei were stained with Hoechst-33342 (1:10000, GeneCopoeia, United States). Fluorescent signals were visualized under a confocal laser microscope.

### Western Blotting

Rats were killed after each treatment, the hippocampus was collected, and total proteins were extracted using radioimmunoprecipitation assay lysis buffer (Beyotime Biotechnology, China). Protein concentrations were determined by BCA protein assay (Beyotime Biotechnology). Brain tissue extracts from each group were boiled for 5 min and the denatured proteins (40 μg) were separated by 10% sodium dodecyl sulfate-polyacrylamide gel electrophoresis and transferred onto nitrocellulose membranes (Bio-Rad, Hercules, CA, United States). After incubation in blocking buffer (0.1% Tween-20 and 5% non-fat-dried milk in Tris-buffered saline) at RT for 1 h, the membranes were incubated with anti-C3 antibody (1:500; Santa Cruz Biotechnology), anti-Iba-1 antibody (1:1000; Sigma), or anti-β-actin antibody (1:2000; Zhuangzhi). Semi-quantitative analysis of protein expression was carried out using horseradish peroxidase-conjugated secondary antibodies (1:5000; Zhuangzhi) and an electrochemiluminescence system (Bio-Rad).

### Statistical Analysis

Fluorescent areas were measured using Image-Pro Plus 6.0 software. All statistical analyses were carried out using SPSS17.0 software and presented as mean ± standard deviation (SD) using GraphPad Prism 6.0 software. Data were analyzed by one-way ANOVA, followed by least significant difference tests for comparisons of three or more samples. Statistical significance was set at *P* < 0.05.

## Results

### Cell Morphology of A1 and A2 Astrocytes

**FIGURE 1:**
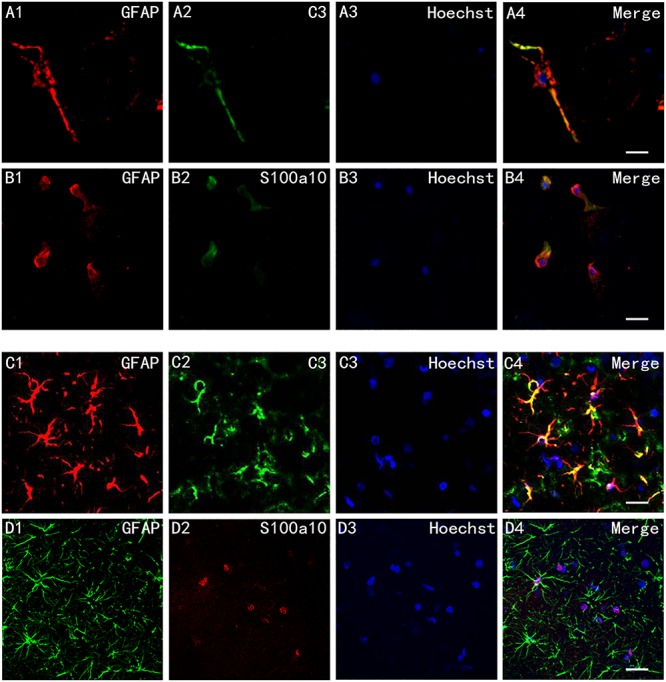
Cell morphology of A1 and A2 astrocytes *in vitro* and *in vivo*. **(A1–A4)** Immunofluorescence staining of cultured astrocytes *in vitro* after exposure to infrasound for 2 h with glial fibrillary acidic protein (GFAP) and component 3 (C3), and **(B1–B4)** with GFAP and S100a10. **(C1–C4)** Immunofluorescence staining of astrocytes in the CA1 region in rats after exposure to infrasound for 3 days with GFAP and C3, and **(D1–D4)** with GFAP and S100a10. Nuclei stained with Hoechst. Scale bar: 25 μm.

We cultured rat hippocampus astrocytes *in vitro* and exposed them to infrasound of 16 Hz and 150 dB for 2 h, followed by immunofluorescence staining with S100a10, C3, and GFAP. Fluorescence microscopy showed that C3^+^ A1 astrocytes had ob- vious long dendrites (Figure 1A), while S100a10^+^ A2 astrocytes had hypertrophic cell bodies with few dendrites (Figure 1B).

*In vivo*, we pretreated rat by infrasound with a frequency of 16 Hz and a pressure level of 150 dB. Double-immunofluorescence labeling with S100a10, C3, and GFAP was also carried out on brain slices of the rat. Immunofluorescent staining with C3 and GFAP highlighted both the cell bodies and slender processes of A1 astrocytes (Figure 1C). But immunofluorescent staining with S100a10 only highlighted the cell bodies of astrocytes (Figure 1D).

### Infrasound Induced A1 Astrocytes

**FIGURE 2:**
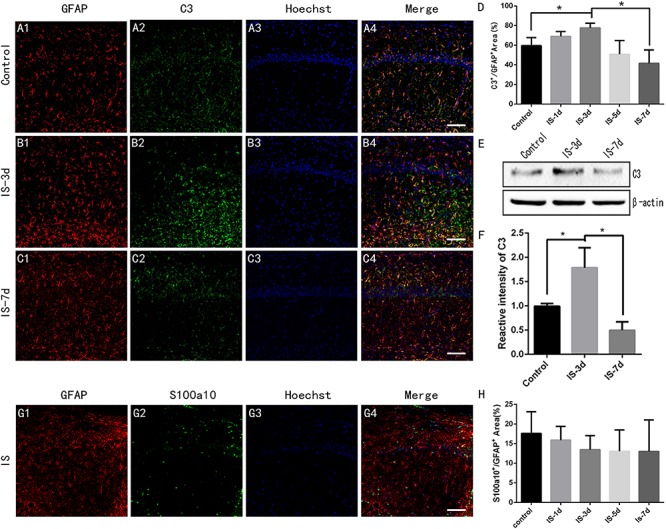
A1 astrocytes were induced in rat hippocampus after 3 days of infrasound exposure. **(A1–A4)** A1 astrocytes in the CA1 region of the rat hippocampus double-labeled by C3 and GFAP in the control group without infrasound exposure, **(B1–B4)** in the group exposed to infrasound (IS) for 3 days (IS-3d), and **(C1–C4)** in the group exposed to infrasound for 7 days (IS-7d). **(D)** Quantitative analysis of percentage of C3^+^/GFAP^+^ area in the CA1 region of rat hippocampus. **(E)** C3 expression in rat hippocampus in control group, IS-3d group, and IS-7d group, by western blotting. **(F)** Semi-quantitative analysis of C3 expression in rat hippocampus in control group, IS-3d group, and IS-7d group. **(G1–G4)** A2 astrocytes in the CA1 region of rat hippocampus double-labeled by S100a10 and GFAP. **(H)** Quantitative analysis of percentage of S100a10^+^/GFAP^+^ area in the CA1 region of rat hippocampus. Nuclei stained with Hoechst. Scale bar: 100 μm. *N* = 6–8 for each experiment. One-way ANOVA. Values are all expressed as mean ± SD. *P < 0.05.

Then, we used double-immunofluorescent labeling to investigate the effects of infrasound on the activation states of the two types of astrocytes in the CA1 region of the rat hippocampus. The percentage of C3^+^/GFAP^+^ area increased after exposure to infrasound for 3 days (*P* < 0.05) (Figures 2A–D). Previous studies demonstrated that GFAP expression increased after infrasound damage (Shi et al., 2013, 2018), and we therefore concluded that A1 astrocytes were activated by infrasound exposure. We also analyzed C3 protein expression levels in rat hippocampus semi-quantitatively by western blotting and confirmed that C3 protein expression levels in the hippocampus were up-regulated after 3 days of infrasound exposure (*P* < 0.05) (Figures 2E,F).

There were no significant differences in the percentage of S100A10^+^/GFAP^+^ area between control rats and rats exposed to infrasound for 1, 3, 5, or 7 days (*P* > 0.05) (Figures 2G,H). These results suggested that infrasound induced A1 astrocytes, but not A2 astrocytes.

### Microglial Cells Were Reactive Following Infrasound Exposure

**FIGURE 3:**
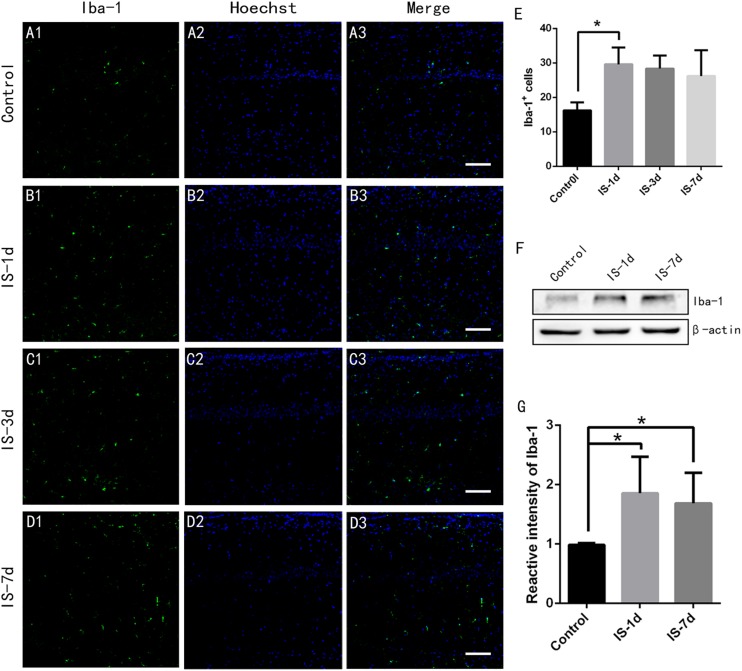
Microglial cells in rat hippocampus increased after 1 day of infrasound exposure. **(A1–A3)** Microglial cells in the CA1 region of the rat hippocampus labeled with Iba-1 in the control group without infrasound exposure, **(B1–B3)** in the IS-1d group (1 day infrasound exposure), **(C1–C3)** IS-3d group (3 days infrasound exposure), and **(D1–D3)** in the IS-7d group (7 days infrasound exposure). **(E)** Quantitative analysis of Iba-1^+^ cells per 625 μm × 625 μm in the CA1 region of the rat hippocampus by immunofluorescence staining. **(F)** Iba-1 expression in rat hippocampus in the control group, IS-1d group, and IS-7d group by western blotting. **(G)** Semi-quantitative analysis of Iba-1 expression in rat hippocampus in the control group, IS-1d group, and IS-7d group by western blotting. Nuclei stained with Hoechst. Scale bar: 100 μm. *N* = 6–8 for each experiment. One-way ANOVA. Values are all expressed as mean ± SD. *P < 0.05.

Liddelow et al. (2017) demonstrated that reactive microglia induced A1 astrocytes, and we therefore determined if microglial cells were activated by infrasound. We labeled microglial cells by Iba-1 immunofluorescence. The results showed that the number of Iba-1^+^ microglial cells in the CA1 region of the rat hippocampus increased after 1 day of infrasound exposure (*P* < 0.05) (Figures 3A–E), consistent with the results of a previous study (Xu et al., 2008). Western blotting confirmed that Iba-1 expression in the rat hippocampus increased after 1 day of infrasound exposure. Microglial cells decreased slightly during 1 day and 7 days, but the result was not significant (*P* > 0.05) (Figures 3E–G). These results suggested that infrasound exposure for 1 day activated microglial cells in the rat hippocampus.

### A1 Astrocytes Were Regulated by the FGF2/FGFR1 Pathway

**FIGURE 4:**
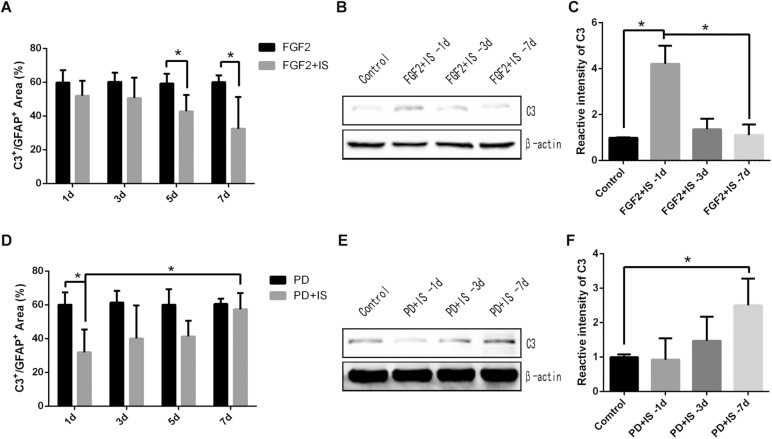
A1 astrocytes were gradually down-regulated by activation of the FGF2/FGFR1 pathway and were up-regulated by inhibition of the FGF2/FGFR1 pathway. **(A,D)** Quantitative analysis of percentage of C3^+^/GFAP^+^ area in the CA1 region of rat hippocampus in different groups, by immunofluorescence staining. **(B,E)** C3 expression in rat hippocampus in different groups by western blotting. **(C,F)** Semi-quantitative analysis of C3 expression in rat hippocampus in different groups. *N* = 6–8 for each experiment. One-way ANOVA. Values expressed as mean ± SD. *P < 0.05.

We initially investigated the role of the FGF2/FGFR1 pathway in A1 astrocyte reactivity by pretreating rats with FGF2 and then exposing them to infrasound. Double-immunofluorescent labeling revealed that the percentage of C3^+^/GFAP^+^ area in the CA1 region of the rat hippocampus were decreased during 1 and 7 days of FGF2 treatment and infrasound exposure (*P* < 0.05) (Figure 4A). Expression levels of C3 in the rat hippocampus also decreased during 1 and 7 days of treatment, as demonstrated by western blotting (*P* < 0.05) (Figures 4B,C).

We then pretreated rats with PD173074 to inhibit activation of the FGF2/FGFR1 pathway. In the CA1 region of the rat hippocampus, the percentage of C3^+^/GFAP^+^ area was increased during 1 and 7 days of PD173074 treatment and infrasound exposure (*P* < 0.05) (Figure 4D). C3 expression levels increased gradually as well (*P* < 0.05) (Figures 4E,F). In our previous research (Shi et al., 2018), GFAP positive cells were not affected by the FGF2 or PD173074 treatment. Overall, these results suggested that A1 astrocytes were gradually down-regulated by activation of the FGF2/FGFR1 pathway and were up-regulated by inhibition of the FGF2/FGFR1 pathway after infrasound damage.

## Discussion

Astrocytes are the main type of glial cells in the brain and spinal cord. Astrocytes undergo astrogliosis during CNS injury and eventually transform into reactive astrocytes (Zamanian et al., 2012; Anderson et al., 2014), as demonstrated for A1 astrocytes in spinal cord injury, CNS brain trauma, and neuroinflammatory and neurodegenerative diseases (Liddelow et al., 2017). Furthermore, we previously demonstrated that infrasound exposure also induced astrocyte and microglial activation (Shi et al., 2013; Cai et al., 2014).

In this study, we examined the activation of microglia and astrocytes to investigate the mechanisms responsible for infrasound-induced neuronal apoptosis in the hippocampus. C3^+^ and GFAP^+^ A1 astrocytes in the CA1 region of the rat hippocampus were activated after 3 days of exposure to infrasound, while Iba-1^+^ microglial cells were activated after 1 day of exposure, suggesting that microglial cells were activated before A1 astrocyte reactivity. Infrasound-activated microglia have previously been shown to produce a wide range of proinflammatory cytokines, including interleukin (IL)-1β, IL-6, IL-18, and tumor necrosis factor (TNF)-α (Cai et al., 2014). Furthermore, Liddelow et al. (2017) indicated that microglia-derived IL-1α, TNF, and C1q worked together to mediate A1 astrocyte reactivity, while A1 astrocytes exerted powerful neurotoxic effects that killed CNS neurons. Overall, these findings suggest that infrasound exposure initially activated microglial cells in the hippocampus, with subsequent induction of A1 astrocytes and neuronal cell apoptosis, resulting in learning and memory impairments.

The current results also found that FGF2 could restrain the reactivity of A1 astrocytes in the hippocampus after infrasound exposure. This neuroprotective effect of FGF2 against infrasound damage was in accord with previous studies showing that FGF2 could regulate the ability of the newborn hippocampal dentate gyrus, the formation of functional circuits, and the structural plasticity of neurons (Kirby et al., 2013; Pollak et al., 2014). Given that FGF2 could trigger FGFR1 signaling (Sutthiwarotamakun, 2011) and the FGF2/FGFR1 pathway may exert an inhibitory effect on astrocyte-mediated inflammation (Shi et al., 2018), we deduced that the neuroprotective effect involved the inhibition of astrocyte-mediated inflammation via the FGF2/FGFR1 pathway. In contrast, we observed that A1 astrocytes gradually increased in the hippocampus during 7 days of infrasound exposure with PD173074 treatment, thus confirming that inhibition of the FGF2/FGFR1 passageway caused reactivity of A1 astrocytes.

This study had several limitations. We used the method of Liddelow et al. (2017) in the immunofluorescence experiment, but we could not provide enough evidence to verify Liddelow et al.’s (2017) conclusion that the A2-specific marker S100a10 did not co-localize with C3 positive A1 astrocytes. In our experimental results, we found C3 existed in most of astrocytes which were labeled by GFAP (Figures 2A–C). It is possible that C3 exist in brain of normal animals (Rong et al., 2007; Fonseca et al., 2011). C3-positive astrocytes is expressed at a low level in normal physiological state, and some brain injury factors may lead to increased pathological expression, thus resulting in neurotoxic effects (Liddelow et al., 2017). We focused on the neurotoxic function of A1 astrocytes in infrasound damage; however, infrasound is a type of noise and might thus also have psychological effects. Furthermore, it is possible that pathways other than the FGF2/FGFR1 pathway might be involved in regulating the reactivity of A1 astrocytes. Further research is therefore needed to identify other potential mechanisms involved in the neurotoxic function of A1 astrocytes in infrasound damage.

In summary, our results demonstrated that A1 astrocytes were induced by microglia in the CA1 region of the rat hippocampus after infrasound exposure, and that these A1 astrocytes were regulated by the FGF2/FGFR1 pathway. Inhibiting A1 astrocytes or activating the FGF2/FGFR1 pathway may thus represent promising targets for the treatment of infrasound-induced CNS injury.

## Ethics Statement

All procedures used in this study were approved by the Institutional Review Board and were performed according to the Guidelines of Institutional Animal Care and Use Committee at the Fourth Military Medical University.

## Author Contributions

All authors contributed substantially to this work. LM conceived and designed the experiments. L-HZ performed the experiments and drew the figures. Y-JS and HH analyzed the data. S-MJ reviewed the language and grammar and provided reference materials. F-FH contributed reagents and analysis tools. X-MW and FW searched and provided material for writing the manuscript, and arranged the manuscript in accordance with the journal specifications. L-HZ, Y-JS, and HH wrote the manuscript.

## Conflict of Interest Statement

The authors declare that the research was conducted in the absence of any commercial or financial relationships that could be construed as a potential conflict of interest.
